# Supplementary material for: The evolution and co-evolution of a primary care cancer research network: From academic social connection to research collaboration
Source: PLoS One. 2022 Jul 29;17(7):e0272255. doi: 10.1371/journal.pone.0272255 (PMC9337668; doi:10.1371/journal.pone.0272255)
Supplement: S1 Appendix — (DOCX) [file pone.0272255.s001.docx]

| **Which other members of the CanTest family do you know professionally, and how did/do you connect and interact with them?***Select 'yes' for all the columns that apply by clicking the drop-down arrow;* leave the box blank for ‘no’ or ‘not applicable’ | | | | | | | | |
| --- | --- | --- | --- | --- | --- | --- | --- | --- |
|  | Exchanged emails or WhatsApp messages | | Met at a conference, workshop, meeting or training event NOT organised by CanTest, e.g. Ca-PRI | | Involved in the same individual project (may be multi-institutional) | | At same Institution / Department | |
| Name1 | Yes | - | Yes | - | Yes | - | Yes | - |
| Name2 | Yes | - | Yes | - | Yes | - | Yes | - |
| Name3 | Yes | - | Yes | - | Yes | - | Yes | - |
| … | Yes | - | Yes | - | Yes | - | Yes | - |

**S1 Appendix – Survey: matrix question and informed consent procedure**

1. **Matrix question**
2. **Informed consent procedure**
   1. **Introductory e-mail text**

Dear member of the CanTest family,

We are starting an exciting new project within the CanTest Collaborative and would like to ask for your help. CanTest is funded by Cancer Research UK’s first ever Catalyst award and aims to support capacity building and collaboration in cancer detection research in primary care.

Our new project aims to understand and demonstrate how well CanTest is achieving its goal of strengthening and developing the Primary care cancer research community and network. The project is being run jointly by two of the nine CanTest institutions (Utrecht and Cambridge) and has a steering group consisting of …, …. and …. from the CanTest Senior Faculty.

CanTest started on 1st April 2017 and, since that time, you have become a member of the CanTest family. We would like you to answer some questions in a brief online survey so that we can retrospectively determine the baseline for the CanTest Network and also the current status of the Network.

This survey asks questions about which other members of the CanTest family you were/are connected with and how you interact with them.

In the short term, we would like to follow up on this survey with questions about how you collaborate with those members of the CanTest family for which you have indicated a connection and also to ask about further connections you may make. In addition, at several points in the future, we will contact you to ask further questions so that we can see how the network changes and grows in time.

The success of this project relies on as many of you as possible taking part, so we would very much appreciate it if you would take the time to participate in this project both now and in the future.

So, please reflect on your connections to CanTest and click the link below to complete the survey.

**[link]**

The survey can be a little slow to load on some pages, due to the fact that we ask network questions, so please be patient.

Please don’t hesitate to contact the core project team if you have any questions or concerns.

…..

…..

…..

- 1. **Introduction page of the survey**

Introduction

CanTest is funded by Cancer Research UK and aims to support capacity building and collaboration in the international cancer detection research in primary care community. This project aims to understand and demonstrate how well CanTest is achieving its goal of strengthening and developing the primary care cancer research community and network.

The results of this survey will be used to map the relationships between the researchers directly associated with CanTest. Analysis will be carried out to determine how these relationships contribute to the goal of building a network of researchers in primary care cancer research**.** Social Network Analysis methodology will be applied for this analysis of the CanTest network.

Completion of the survey should take less than 15 minutes of your valuable time. Participation does not contain any risks.

Use and storage of data

All information gathered in this study is strictly confidential. All data will be kept in a locked file cabinet and password protected computer. At any point during the research, you are free to opt-out and your data will not be used after that time.

If you require further information

If you have any questions – before, during or after the study – you can contact the survey researcher through the contact information provided below.

……

- 1. **Informed consent question**

| Yes | No |
| --- | --- |

I have been sufficiently informed about this study, and accordingly I agree to participate in this survey.
