# Supplementary material for: The evolution and co-evolution of a primary care cancer research network: From academic social connection to research collaboration
Source: PLoS One. 2022 Jul 29;17(7):e0272255. doi: 10.1371/journal.pone.0272255 (PMC9337668; doi:10.1371/journal.pone.0272255)
Supplement: S2 Appendix — (DOCX) [file pone.0272255.s002.docx]

**S2 Appendix – Effects used in the three models**

|  |  | **Evolution** | |  | **Coevolution** |
| --- | --- | --- | --- | --- | --- |
|  |  | **Model 1**  Academic social network | **Model 2**  Collaborative network |  | **Model 3**  Academic social network → Collaborative network |
| **Within networks** | |  | |  |  |
| **Individual researcher’s positions** | |  | |  |  |
| **Outdegree/density** | **Figure 2a**: tendency of researchers to create or terminate relationships with others independent of other processes^1^ |  | X |  | X |
| **Reciprocity** | **Figure 2b**: tendency of researchers to reciprocate relationships^2^ | X |  |  |  |
| **Transitivity** | **Figure 2c**: tendency of researchers to have relationships in small dense clusters in the network (network embeddedness) | X | X |  | X |
| **Indegree-popularity** | **Figure 2d:** tendency of researchers who have many incoming relationships to receive more incoming relationships over time (preferential attachment) | X |  |  |  |
| **Outdegree-popularity** | **Figure 2e:** tendency of researchers who have many outgoing relationships to receive more incoming relationships over time | X |  |  |  |
| **In=outdegree popularity/activity** | **Figure 2d,e:** combination of the above-explained degree popularity and the degree activity^3^ |  | X |  | X |
| **Out/in degree assortativity** | **Figure 2f:** tendency of researchers with many relationships to be related |  | X |  | X |
| **Truncated outdegree** | **Figure 2g:** tendency of researchers with less than *x* outgoing relationships to create new relationships | X |  |  |  |
| **Network isolate** | **Figure 2g:** tendency of researchers with zero relationships to create relationships |  | X |  | X |
| **Individual researcher’s characteristics** | |  |  |  |  |
| **Ego** | **Figure 2h:** tendency of researchers with higher values for a related characteristic (e.g. higher seniority) to create more outgoing relationships | X |  |  |  |
| **Alter** | **Figure 2i:** tendency of researchers with higher values for a related characteristic (e.g. higher seniority) to receive more incoming relationships | X |  |  |  |
| **Ego+alter** | **Figure 2h,i:** combination of the above-explained ego and alter effect^3^ |  | X |  | X |
| **Same/similarity** | **Figure 2j:** tendency of researchers to have relationships with other researchers that are similar to themselves (e.g. researchers of similar gender) | X | X |  | X |
| **Between networks** |  |  |  |  |  |
| **Direct effect (‘dyad-level’)** | **Figure 2k:** tendency of researchers to have a relationship in one network if they also have that relationship in the other network (entrainment effect) |  |  |  | X |
| **Degree effects (‘actor-level’)** | **Figure 2l:** tendency of researchers to have many (incoming/outgoing) relationships in one network if they also have many relationships in the other network |  |  |  | X |
| **Agreement effect (‘triad-level’)** | **Figure 2m:** tendency of researchers to have the same relationships in one network as those of the researchers they are related to in the other network |  |  |  | X |
| **Interactions** |  |  |  |  |  |
| **Ego x ego** | Tendency of researchers with higher attribute values (e.g. gender and seniority) to have more relationships | X | X |  | X |
| **Transitivity x reciprocity** | Tendency of researchers to reciprocate relationships in clusters | X |  |  |  |
