# Supplementary material for: The evolution and co-evolution of a primary care cancer research network: From academic social connection to research collaboration
Source: PLoS One. 2022 Jul 29;17(7):e0272255. doi: 10.1371/journal.pone.0272255 (PMC9337668; doi:10.1371/journal.pone.0272255)
Supplement: Attachment — Submitted filename: Response to Reviewers.docx [file pone.0272255.s003.docx]

*The evolution and co-evolution of a primary care cancer research network: from academic social connection to research collaboration*

Dear editor, dear mr. Bianchi,

Thank you for the invitation to submit a revised manuscript. We have very carefully considered the constructive feedback received from the reviewers and have incorporated their comments in the revised version of our manuscript. For your convenience, we first reproduce the reviewers’ feedback before detailing our responses. The opportunity to respond to this feedback has helped us to improve the clarity of our work, for which we are grateful. We trust that these revisions are satisfactory; if not, please let us know which further points need clarification and adjustment.

With kind regards,

Debbie Vermond, Esther de Groot, Valerie A. Sills, Georgios Lyratzopoulos, Fiona M. Walter, Niek J. de Wit, Greg Rubin

**Reviewers’ comments to authors**

**Reviewer 1**

We are thankful for the reviewer’s suggestions to improve the comprehensibility of our manuscript.

|  | **Reviewer’s feedback** | **Our response** |
| --- | --- | --- |
| **1.** | Abstract:   - It is desirable that a brief summary of the conclusions be described in the abstract to inform the reader if the objectives of this work have been achieved. | We recognize that the conclusions were not very clear from our abstract, presumably because there was a difference in terminology used to describe the objectives and the conclusions in the abstract. We adjusted the part of the abstract presenting the conclusions accordingly (changes in bold):  Abstract, page 2 , line 33-40 (in the file 'Revised Manuscript with Track Changes'):   - *“Visualizing the development of the CanTest network revealed that researchers within CanTest get to know each other quickly and also start collaborating over time* ***(evolution of the academic social network and collaborative network respectively)****. Results point to a stable and solid academic social network that is particularly encouraging towards more junior researchers****; yet differing for male and female researchers (the effect of individual researcher characteristics)****.* ***Moreover,*** *although the academic social network and the research collaborations do not grow at the same pace, the benefit of creating academic social relationships to stimulate effective research collaboration is clearly demonstrated* ***(co-evolution of both networks)****.”* |
| **2.** | Abstract:   - Authors need to standardize the format of the word “co-evolution”. In the text there are several formats: “co-evolution”, “(co-)evolution” and “coevolution”. | Thank you for your attention to detail and careful reading. We have adapted the manuscript accordingly such that we exclusively use the version ‘co-evolution’ and not ‘coevolution’. Where we used the format ‘(co-)evolution’, we now use ‘evolution and co-evolution’. |
| **3.** | Introduction, second paragraph:   - The presentation of “The Can Test Collaborative (CanTest)” is not clear. It is important to introduce the policy, vision and mission of this working group. It is important to inform the website address of the CanTest. - The text describes that academic centers in five different countries participate in CanTest. It is important to describe the name of the institutions and countries. - May institutions interested in participating in this working group submit an application? - What have been the results obtained by CanTest since its creation in 2017, for example: number of projects developed, articles published, organization of events, research exchange program. | We agree with the reviewer that the manuscript could provide more clarity on the CanTest collaborative as a case used in in this manuscript. At the same time, however, the aim of this manuscript was not to present or promote CanTest as a collaborative, but rather to use CanTest as a case study to illustrate how research networks may evolve and what are its success factors.  Accordingly, we tried to find a balance in providing more information about CanTest to improve understanding of the context while keeping this additional information short so as not to overwhelm with too many details. We are happy to offer an Appendix with the details asked for in case the editor of PloS One thinks that this is required. We addressed the comments as such (additions in bold):  Introduction, page 3, line 54-55:   - The CanTest Collaborative (CanTest) is a clear example of such a network **and serves as case study for this manuscript (https://cantest.org)**.   Introduction, page 3, line 57-63:   - It comprises nine academic centres in five different countries and across three continents; **individual researchers from 10 other academic centres are also involved by invitation**. - **Its main objectives are to increase capacity and sustainability of research into early detection and diagnosis of cancer – recruiting and supporting the development of a new generation of researchers to establish themselves – and to assess and evaluate approaches to improving early detection and diagnosis of cancer in primary care (the work carried out in this study will shed insight in how CanTest has addressed the first objective).** |
| **4.** | Introduction, fifth paragraph:   - Was the definition of the term “co-evolution” described in the text obtained from any reference or it is a definition of the authors themselves? The article #35 does not mention this word and it also does not define the term “co-evolution” for the context used in this work. | Thank you again for your attention to detail. The term co-evolution is an existing term and commonly used in social network analysis literature to describe the interdependent dynamics of two or more networks comprising the same set of people (in contrast to ‘evolution’, which refers to the development and dynamics of single networks). We have added a reference to refer to this concept accordingly:  *Snijders, Tom & Lomi, Alessandro & Torlo, Vanina. (2013). A model for the multiplex dynamics of two-mode and one-mode networks, with an application to employment preference, friendship, and advice. Social networks. 35. 265-276. 10.1016/j.socnet.2012.05.005.* |
| **5.** | Introduction, fifth paragraph:   - Review the work objectives: “In this study, we aim to increase knowledge on (1) the evolution of academic social networks and collaborative networks and the influence of individual researcher characteristics and positions, and (2) the co-evolution of the academic social network and the collaborative network.” | We have reviewed our objectives and reformulated them as following:  Introduction, page 5, line 107-111:   - The objectives of this study are to increase knowledge on (1) the evolution of academic social networks and collaborative networks, (2) the co-evolution of academic social networks and collaborative networks, and **(3) the influence of individual researcher characteristics and their network positions on evolution and co-evolution.** |
| **6.** | Introduction, fifth paragraph:   - It is not clear if any other working group, similar to CanTest, participated as partnership in this research. | No other working group was involved. |
| **7.** | Introduction, fifth paragraph:   - The text comments that this work will evaluate individual researcher characteristics and positions. What would be these influences? | In row 5 above, we reformulated our objectives such that the role of individual characteristics and network positions in our study was presented more clearly. By the unique link between a network of academic social relationships and a network of collaborative relationships, we could study how researcher’s characteristics and their network positions influence the (concurrent) development of such networks.  The sentence saying that researcher characteristics and positions may play a role in network development was perhaps too ‘hidden’ as a second sentence of the third paragraph:  *“Many factors may play a role in the evolution of relationships within academic social networks, including both individual characteristics of researchers and their positions in the networks.”*  Accordingly, in addition to our sharpening of the objectives, we have rewritten the third and fourth paragraph such that our interest in the role of individual researcher characteristics and their network positions follows more clearly:  Introduction, page 4, line 72-78:   - “However, academic social networks do not necessarily translate into effective collaborative research networks (networks of researchers connected by collaboration); **individual characteristics of researchers in networks are considered to play a significant role in their development [15-17].** Two characteristics of researchers that have been thoroughly studied are gender and seniority [20, 21]. […..]”   Introduction, page 4, line 86-88:   - **“Another factor that may play a prominent role in explaining the development of networks is individual network positions [18, 19]. Along with the individual characteristics of researchers, their positions in relation to each other may steer their relationships.** [….]**”** |
| **8.** | Methods:   - Attach the form template that was used for the survey. Was this form validated before application? - Attach the model of informed consent. - The description of the matrix question is confusing and difficult to understand. It is suggested to build a table with the questions and possible answers. | We have added the matrix question as an appendix to the manuscript (S1 Appendix). We hope that this is sufficient to make the description of the question and possible answers easier to understand. With regard to the question of validation of this matrix: prior to sending the network survey to the CanTest researchers, the face validity of the survey was assessed by a group of native speakers. They reviewed the surveys for ease of use and understanding. All of them deemed the network survey to be acceptable. We have added this to the manuscript accordingly:  Methods, page 7, line 151-154:   - **“Prior to sending the network survey to the CanTest researchers, the face validity of the survey was assessed by a group of native speakers. They reviewed the survey for ease of use and understanding. All of them deemed the network survey to be acceptable.”**   The use of a matrix, or roster, is customary practice in network research to question the connections in a network. We realize that we had not adequately explained this, so we have now added some additional explanation on the use of a matrix question and a reference on this method of data collection to the methods section of our manuscript:  Methods, page 6-7, line 138-146:   - “**As is customary in network research,** one matrix question, or roster, explored the relationships between researchers in the CanTest network at three points in time [….] **[Butts, 2008]*****.** **From this roster of all researchers in the network,** **researchers were asked to identify other researchers with whom they were connected.** For each of the 60 researchers in the network, they selected either ‘yes’ or ‘no’ for any of four possible ways of being connected: [….] **(S1 Appendix)**”.   * *Butts, Carter. (2008). Social Network Analysis: A Methodological Introduction. Asian Journal of Social Psychology. 11. 13 - 41. 10.1111/j.1467-839X.2007.00241.x.*  Furthermore, regarding the informed consent form: informed consent was dealt with by providing study information in the invitation e-mail and survey introduction, after which the first survey question was: “*I have been sufficiently informed about this study, and accordingly I agree to participate in this survey (yes/no)*”. Only if the answer to this question was yes, the survey became available. Along with the matrix question itself, we have added the invitation e-mail and the survey introduction to S1 Appendix and referred to that in the manuscript:  Methods, page 6, line 138:   - “Informed consent was obtained at the start of each survey **(S1 Appendix)**.” |
| **9.** | Results:   - Table 1: The definitions could be described as a label below the table. Each item in the table could be better explained in the text or in the label text. - Table 3: Write a label for the words “par.” e “(s.e.)”. - What were the time intervals T0, T1 and T2 in tables 1 and 2? | We have adapted Table 1 such that its label provides an interpretation of the numbers reported:   - **“^1^ An average outdegree of 9 means that researchers know on average 9 other researchers; ^2^ An s.d. outdegree of 6.8 means that on average the difference between the average outdegree and individual measurements is 6.8; ^3^ A reciprocity of 0.65 means that 65% of the connections between researchers are mutual; ^4^ A transitivity of 0.51 indicates that 51% of the connections were transitive (referring to network embeddedness/transitivity).”**   Initially, we also moved the definitions from the second column to the label of the table, yet, for reasons of clarity, we decided to move them back to the table itself. Without the definitions directly at hand, it seemed more difficult to understand the numbers reported.  Furthermore, the label of Table 3 was adapted such that “par” and “(s.e.)” were written fully: **“par. = parameter for the effect (estimate); (s.e.) = standard error”**.  Finally, the time intervals for T0, T1 and T2 in Table 1 and 2 were explicated in the methods section. To repeat the explanations in the Tables made them more cluttered. We trust that this is satisfactory, but should you have any further concerns, please let us know.  *“Data on the academic social relationships between all 60 researchers in the CanTest network at the time of this study were collected through online surveys at three points in time:*   - *T0 - Point of entry to CanTest (April 2017/2018) – before the first International School* - *T1 - April 2019 – just before the second International School* - *T2 - June 2019 – just after the second International School.”* |
| **10.** | Results:   - I suggest a brief explanation of the Jaccard coefficient and how this coefficient is interpreted. Could these data be inserted in table 1? | The Jaccard similarity index is a measure of similarity between two sets of data – where higher numbers indicate higher similarity.  Jaccard Similarity = (number of connections in both networks) / (number in either network)  We have added this information to the manuscript accordingly (changes in bold):  Results, page 12, line 244-251:   - “In addition, the association at the relationship-level (how many relations between researchers in the academic social network are also present in the collaborative network and vice versa) can be expressed by the Jaccard similarity index. **This is a measure of similarity between two sets of data, formally defined as the number of connections in both networks divided by the number in either network, with higher values being indicative for higher similarity. The Jaccard similarity index for each of the three observations** was 0.19, 0.13, and 0.11. If independence between the networks was assumed, the expected Jaccard coefficients would be 0.03, 0.04, and 0.08. The observed values, while not very high, were higher.”   It is not possible to add these data to Table 1. Table 1 reports separate measures for both networks, where the Jaccard coefficient is a measure of similarity *between* the networks. |
| **11.** | Results:   - In the item "Network co-evolution" I suggest to highlight and explain in the text the most relevant points of the table 3. | Thank you for this suggestion. Throughout the result section, we only presented significant results from the tables in the text. We recognize that the text in the paragraph on ‘Network co-evolution’ is only very short, but, this is because apart from the within-network effects (that were already measured and discussed in the paragraph on ‘Network evolution’) there was only one significant cross-network effect. Thus, we reported that finding in the ‘Network-coevolution’ paragraph and also included its interpretation, yet described that “other cross-network effects were not significant”. |
| **12.** | Discussion and conclusions:   - A point that needs to be discussed is if the CanTest is achieving the desired goals within the context of the policy, vision and mission of this working group. - If the goals are being achieved, what are the strengths and opportunities for improvement. - If the objectives are not being achieved, what are the failures, weaknesses and action plans for continuous improvement of this working group. | In line with comment 3 we would like to stress that CanTest only serves as a case study for our manuscript, and that it was not the initial intention to evaluate how the network analysis impacted the ambitions of Cantest.  In the manuscript, we have added shortly on how CanTest addressed it’s first objective (as added in response to the reviewer’s comment in row 3 above):  Discussion, page 15-16, line 290-294:   - “The CanTest network is clearly successful in connecting researchers with each other. Researchers within the network **establish connections promptly and effectively**, allowing for considerable exchange of information and ideas **to increase capacity and support sustainability of early detection and diagnosis of cancer research**.” |
| **13.** | Discussion and conclusions:   - I suggest to review the conclusions. The text content is not in line with the objectives described in the introduction. | Now that we have reformulated our objectives (row 5), we hope that the coherence of the objectives and the conclusions is clearer. In addition, we now specifically referred back to the objectives in our discussion by adding the objectives in brackets after the relevant results:  Our first objective, to study the evolution of academic social networks and collaborative networks, was addressed in the first two paragraphs (page 15-16, line 290-306):   - “*The CanTest network is clearly successful in connecting researchers with each other. Researchers within the network establish connections between them promptly and effectively* ***(objective 1: evolution of the academic social network)****, allowing for considerable exchange of information and ideas to increase capacity and support sustainability of early detection and diagnosis of cancer research.*” - “*they seem to expand their collaborative research networks more slowly* ***(objective 1 and 3: evolution of the collaborative network and the influence of individual researcher characteristics)****.*”   Our second objective, to study the co-evolution of academic social networks and collaborative networks, was likewise addressed in the second paragraph (page 16, line 306-314):   - “*The positive but decreasing association between the CanTest academic social network and the collaborative network over time further confirms this* ***(objective 2: co-evolution of the academic social network and the collaborative network)****. The concurrent development of effective research collaboration is indeed a time consuming process – being connected informally, e.g. by being involved in the same project, may lead to collaboration on ideas and study design, application for funding, exchange visits, conduct of research, and only finally co-authorship. Despite this lengthy sequence, our results demonstrate that the transition from social connections into research collaboration does take place: creating an academic social relationship between two researchers significantly increases their chances to collaborate.*”   Our third objective, to study the influence of individual researcher characteristics and their network positions on the processes in the first two objectives, was addressed in the first two paragraphs of the discussion as well (page 16, line 294-306):   - “*Seniority and gender seem to play a major role in the development of relationships within the network* ***(objective 3: individual researcher characteristics)****, not affecting who is known or not, but which researchers get to know others* ***(objective 3: individual network positions)****.* *We found that more junior researchers built and expanded their academic network, but that with increasing seniority researchers were less likely to get to know others. This closely aligns with one of the key objectives of CanTest to support the next generation researchers to establish themselves and reach early independence in their academic social connections [11]. Moreover, compared to male researchers, female researchers seem to expand their academic social networks faster. The interaction between gender and seniority stresses even more how males of increasing seniority are less likely to expand their academic social networks over time.*” - “*Yet, in contrast with how more junior researchers expand their academic social networks, they seem to expand their collaborative research networks more slowly* ***(objective 1 and 3: evolution of the collaborative network and the influence of individual researcher characteristics)****.*” |

**Reviewer 2**

We are thankful for the valuable feedback of the reviewer to help improve our manuscript.

|  | **Reviewer’s feedback** | **Our response** |
| --- | --- | --- |
| **1.** | Thank you for the opportunity review this article. I really enjoyed reading it, it is very well written and I think it is worth publishing. I think that the authors can clarify a few points. | Thank you. |
| **2.** | They need to define in very clear terms what they mean by social network (which they later call academic social networks and I think they should use one term all the way through) and a collaborative networks very early on in the publication | We thank the reviewer for the suggestion and have added a box with definitions of the academic social network and the collaborative network to the methods section (Methods, page 8, Box 1).  We defined the academic social network as a “network of researchers connected by informal interactions and social relationships”, and the collaborative network as a “network of researchers connected by co-authorship as a proxy for collaboration”.  We made sure that the introduction now exclusively makes use of the terms ‘academic social networks’ and ‘collaborative networks’ (and no ‘social networks’, for example) to reduce confusion on terminology. Yet, because the introduction was used to sketch the landscape for networks in general, where the definitions for the networks studied in this manuscript (e.g. taking co-authorship as a proxy for collaboration) were only operationalized in the methods, we added the boxes to the methods section. |
| **3.** | More information about distinguishing between the two is required both as concepts and also in their approach to analysis (see point 3 below)  3. The data they use for the social network analysis includes if study participants are ‘involved in the same individual project’. This suggests some tautology as this same project can in fact be something that generates a publication – in fact it would be what one might expect in an academic / research environment. The two are interrelated so how did they distinguish between the two? Further as the data for the SNA are then reduced into a yes/no option this may have a very large influence on the findings.  It may be that my point above is dealt with by the method of analysis that they use ‘ stochastic actor-based models’ – but I doubt it. Someone who is more knowledgeable with SNA would need to comment on this. However, I can’t see how they deal with the overlap of ‘working on the same project’ with ‘publishing’ especially as working in the same project is it is combined with the other markers of the academic social network. I am wondering if repeating the analysis excluding data related to ‘involved in the same individual project’ would produce a different result? | The definitions provided above distinguish academic social networks and collaborative networks conceptually.  The reviewer’s second remark addresses the distinction between the two with respect to the analysis. Being involved in the same individual project may generate a publication, but so may the other three items of the academic social network (exchange of e-mails, meeting at a conference, or working in the same institution/department). However, this overlap between being connected informally (the academic social network) and publishing together (the collaborative network) is not problematic. Actually, the aim of our work is to measure the amount of overlap to find out to what extent one leads to the other: we constructed the data collection such that the four items of the academic social network are markers of self-perceived professional socialization (a process measure), whereas being involved in the same publication is a marker of actual collaboration (an outcome, visible in bibliographic databases). We have added this difference explicitly to Box 1.  Hence, that the two concepts are inter-related is the basis on which our analysis is built. Our analysis highlights how much they are inter-related, considering that (1) some initiatives, although intended to become publications, do not progress, and (2) there are lag phases between professional socialization and publication. Accordingly, the four items, among which ‘being involved in the same individual project’, are not confounders, but potential precursors of joint publications.  With regard to the impact of reducing data into yes/no options: the results of the academic social network data did not provide any discrimination on the four items between groups of researchers and hence gave no rise to the need for separate analyses. Instead, the combination of the four items captured the academic social network in a complete as possible manner: when the answer to any of the four items was yes, the connection was coded as “yes”. Only when the answer to all of the four options was no, was the connection in the academic social network was coded as “no”.  We felt the same need as the reviewer for someone who is more knowledgeable with SNA to comment on this. Our analyses and results were presented to experts: the developers of the stochastic actor oriented model (Tom Snijders and Cristian Steglich). The potential of this model to study the co-evolution of academic social networks and collaborative networks was confirmed.  We adjusted the introduction, methods and discussion section to improve clarity on this point:  Introduction, page 5 , line 100-103 :   - Yet, although academic interaction is considered an important factor in collaboration, we still have limited understanding of how the expansion of academic social networks translates into effective research collaboration**, i.e. to what extent one leads to the other** (the ‘co-evolution’ of two networks)   Methods, page 9, line 199-203:   - Model 3 captures the co-evolution of both networks, exploring the influence of the academic social network on the collaborative network **(to measure how much both networks are inter-related, considering that (1) some initiatives, although intended to become publications, do not progress, and (2) there are lag phases between informal interaction and publication)**   Discussion, page 16, line 309-312:   - […] The concurrent development of effective research collaboration is indeed a time consuming process – **being connected informally, e.g. by being involved in the same project, may lead to collaboration on ideas and study design,** application for funding, exchange visits, conduct of research, and only finally co-authorship. |
| **4.** | In the discussion the authors may want to consider the following points:   - They claim that the social networking is important and ‘This closely aligns with one of the key purposes of CanTest, namely to support the next generation researchers to establish themselves and reach early independence’. However this is only the case if there is a very close correlation between the social network and publication output and I am not sure this case is made at the point at which they come to this conclusion. It seems they base this conclusion the results of social networking. I am not suggesting that they can’t come to this conclusion but it seems to me it must be related to the co-evolution argument. | Thank you for your observation. We agree that this claim particularly relates to our findings from the academic social network. Yet, in line with recent discussions about other indicators of scholarship than publishing only, “supporting a new generation” is not necessarily the same as a close correlation between academic social connections and publication output. Development of researchers is not only in co-authoring, but also in building an academic social network of international researchers.  That said, as discussed already, there are reasons to expect that the collaborative network lags behind the academic social network. In the longer run, we expect that the collaborative network will also show the development and growing independence of junior researchers in terms of being well-grounded and starting/having their own collaborations. We further emphasized this argument in our discussion (line 317-322). |
| **5.** | In the discussion the authors may want to consider the following points:   - The authors note that ‘creating an academic social relationship between two researchers significantly increases their chances to collaborate.’ This seems self-evident – it is hard to work with someone without knowing them. I suspect the point being made is that the processes that Can Test set up is instrumental in this. However without a counterfactual – other ways of people meeting (through co-incidental conferences perhaps) may be just as effective. Perhaps this could be tested if the Can Test training events (excluding conferences) was investigated. | Thank you for raising this; this is of great importance for the comprehensibility of our message, and we have updated the manuscript (changes in bold):  Discussion, page 16-17, line 312-320:   - “Despite this lengthy sequence, our results demonstrate that the transition from social connections into research collaboration does take place: creating an academic social relationship between two researchers significantly increases their chances to collaborate. **The existence of the CanTest Collaborative and the structure, events and coordinated communications that go with it, has most likely contributed to the observed results. However, it is not possible to know how the networks studied here would have evolved without the existence of CanTest. The timing of the significant increase in academic social connections since the start of CanTest in April 2017 makes it likely though that CanTest has been instrumental in the process of creating and accelerating informal interactions and social relationships and hence collaboration.**”   Excluding the CanTest training events from our analysis would not create the counterfactual that the reviewer is looking for, since CanTest also boosts connections between researchers aside from the annual International school (e.g. through online events and exchange visits). In addition, nearly all of the CanTest researchers attend the International Schools, and as such we do not have a group of researchers that attends the school and a group that does not. |
| **6.** | In the discussion the authors may want to consider the following points:   - I suspect what I am wondering is if reducing their data to a yes no in the SNA instead of testing each of the four individual markets of being active in a social network should be investigated? | We anticipate that we have provided an answer to this comment in row 3 above, where we explained how the individual four markers of being connected informally were indeed aggregated for the analysis instead of being tested separately.  The word ‘dichotomized’ (used in our methods section to explain this process) may have caused the confusion here. We recognized that ‘aggregate’ is the better word for this and changed it accordingly in the manuscript:  Methods, page 7, line 146-148:   - For the analysis, it was necessary to **aggregate** the results; we did so by recoding any number of ways of being connected **(either by 1, 2, 3 or 4 items)** as 1 = “yes” and recoding the absence of any connection as 0 = “no”. |
